# Suppressed nuclear factor-kappa B alleviates lipopolysaccharide-induced acute lung injury through downregulation of CXCR4 mediated by microRNA-194

**DOI:** 10.1186/s12931-020-01391-3

**Published:** 2020-06-10

**Authors:** Ruidong Chen, Fei Xie, Jie Zhao, Bin Yue

**Affiliations:** 1 The First Department of Pediatric, Cangzhou Central Hospital, No. 16, Xinhua West Road, Yunhe District, Cangzhou, 061000 Hebei Province P.R. China; 2 The Second Department of Pediatric, Cangzhou Central Hospital, Cangzhou, 061000 P.R. China

**Keywords:** Nuclear factor-kappa B, Chemokine receptor type 4, micrRNA-194; acute lung injury, Inflammation

## Abstract

Acute lung injury (ALI) is a highly lethal pulmonary disease that causes edema, hypoxemia and respiratory failure. Recent evidence indicates that nuclear factor-kappa B (NF-κB) plays a crucial role in ALI development. However, the regulatory mechanism of NF-κB on ALI remains enigmatic. In this study, we investigated potential molecular mechanism of NF-κB on ALI induced by lipopolysaccharide (LPS). BALB/c mice were subjected to intratracheal spraying of LPS to generate an ALI mode, with the activity of NF-κB in mice tissues being detected by enzyme linked immunosorbent assay (ELISA), and the number of inflammatory cells in bronchoalveolar lavage fluid being counted. Then, the macrophage cell line RAW264.7 exposed to LPS were treated with ammonium pyrrolidinedithiocarbamate (PDTC) (inhibitor of NF-κB), miR-194 mimic, or oe-chemokine receptor type 4 (CXCR4) separately or in combination. After that, ELISA and reverse transcription quantitative polymerase chain reaction (RT-qPCR) were used to detect the expression level of IL-1β, IL-6, TNF-α, miR-194 and CXCR4, respectively. In addition, the targeting relationship between miR-194 and CXCR4 was verified by dual-luciferase reporter gene assay. The dry/wet ratio of lung and the MPO activity were also measured to assess the inflammatory response in mice. Activation of NF-κB down-regulated the miR-194 expression in LPS-induced ALI. Overexpression of miR-194 alleviated LPS-induced ALI and reduced the expression of inflammatory factors IL-1β, IL-6 and TNF-α via targeting CXCR4. In LPS-induced ALI, NF-κB mediates the CXCR4 expression by inhibiting the expression of miR-194, thus promoting the inflammatory injury of lung.

## Introduction

Acute lung injury (ALI) is a major cause of acute respiratory distress syndrome (ARDS) which often leads to fulminant respiratory failure and death [1]. It is characterized by pulmonary infiltrates, hypoxemia, as well as damages to the vascular endothelium and lung alveolar epithelium [2]. Causes of ALI include, but are not limited to, severe sepsis linked to a pulmonary or non-pulmonary source, toxic inhalation, lung contusion, acute pancreatitis, burn injury, and cardiopulmonary bypass surgery [3]. Although great efforts have been made in understanding the pathophysiology of ALI, the existing therapies have not diminished the mortality or improved the life quality of survivors [4]. Therefore, it is urgent for us to explore more effective medications and innovative therapies for the ALI.

Acting as one of the transcription factors, nuclear factor-kappa B (NF-κB) was named for its binding with the enhancer element of the immunoglobulin kappa light-chain of B cells [4]. It is a significant inflammatory inducible factor, which mediates the inflammatory response via regulating the transcription of a variety of proinflammatory cytokines, chemokines and adhesion molecules [5]. For instance, punicalagin suppresses lipopolysaccharide (LPS)-induced neuroinflammation, oxidative stress and memory impairment through blockage of NF-kB activation [5]. Furthermore, a prior study has demonstrated that when the NF-κB signaling pathway is activated, the released inflammatory cytokines and chemokines such as IL-1β, IL-6 and TNF-α would execute crucial effects on the progression of ALI [6]. Another study also verifies that NF-κB activation initiated by the IκB-α may accelerate the transcription of IL-1β, IL-6 and TNF-α [7].

On the other hand, microRNAs (miRNAs), as small non-coding molecules, can regulate gene transcription in various biological and pathological processes such as ALI/ARDS, which has become an important field of biomedical research [8]. Extensive studies have been carried out to characterize miRNA expression profile and function in ALI. For example, the down-regulation of miR-7 can ameliorates ALI through elevating its target molecule KLF4, accompanied by altered transduction of the NF-κB signaling pathway [9]. In addition, miR-27a inhibits lung inflammation and cell apoptosis by regulating TLR4/MyD88/NF-κB signaling pathway to alleviates LPS-induced ALI in mice [10]. As one of the miRNAs, miR-194 was known to promote prostate cancer metastasis by inhibiting SOCS2 to activate STAT3 and ERK signaling pathways [11]. Moreover, miR-194 has been identified to relieve inflammatory response through inhibition of the TGF-β/SMAD signaling pathway activation in chronic idiopathic urticarial [12]. Besides, in renal ischemia-reperfusion injury, upregulation of miR-194 can restrain the secretion of proinflammatory cytokines such as IL-1β, IL-6 and TNF-α [13]. However, the regulatory mechanism of miR-194 on inflammatory response of ALI is still unknown. Hence, the purpose of the present study was trying to figure out the potential molecular mechanism for the crucial role of miR-194 mediated by NF-κB on ALI.

## Materials and methods

### Animal model establishment

A total of 48 female BALB/c mice (aged 8 weeks) were chosen and divided into 4 groups. For each group, 12 random mice were treated with LPS only, LPS and ammonium pyrrolidinedithiocarbamate (PDTC) (inhibitor of NF-κB), LPS and dimethyl Sulfoxide (DMSO) or without treatment (control group). ALI was induced by spraying 2 mg/kg LPS (Sigma-Aldrich Chemical Company, St Louis, MO, USA, 1 mg/mL) into the mouse trachea. One hour before LPS treatment, PDTC was intraperitoneally administered to mice at a dose of 50 mg/kg. Also, mice were given intratracheal miR-194 antagomir (15 mg/kg) spray 24 h before LPS treatment. The rate of successful modeling was 100%. Ten mice in each group were selected for the following experiments.

### Bronchoalveolar lavage fluid (BALF) collection and inflammatory cells detection

After 24 h of LPS induction, the right lung of mice was washed twice with 0.7 mL of phosphate buffer saline (PBS) and 1.2 mL of BALF was collected for each sample. Then the collected BALF solution was centrifuged at 4 °C at 150×g for 10 min. Lastly, the obtained cells were resuspended in 500 μL of PBS containing 0.1% bovine serum albumin (BSA) for counting the total cells, macrophages, lymphocytes and neutrophils.

### NF-κB activity measurement

The lung tissue samples were homogenized (1 g of tissue was immersed in 9 mL of saline) and centrifuged at 4 °C for 10 min at 3000×g. The supernatant was collected for testing NF-κB activity via an enzyme linked immunosorbent assay (ELISA) kit (Shanghai Yaji BioTech., Shanghai, China).

### Cell culture and treatment

The macrophages RAW264.7 cell line was purchased from the American Type Culture Collection (Manassas, VA, USA) and cultured in endotoxin-free Dulbecco’s Modified Eagle Medium containing 10% fetal bovine serum (Gibco, Carlsbad, CA, USA). When reaching 80–90% confluence, the cells were treated with 1 μg/mL LPS (strain O55:B5, Sigma Aldrich, St. Louis, MO, USA) alone or together with 25 μg/mL PDTC (inhibitor of NF-κB) or DMSO (as NC), respectively. After 24 h of treatment, the subsequent analyses were carried out.

RAW264.7 cells in logarithmic growth phase were seeded into a 6-well cell culture plate at a density of 4 × 10^5^ cells/well. When the cell confluence reached 80–90%, the cells were transfected with mimic-NC, miR-194 mimic, inhibitor-NC and miR-194 inhibitor (all from Invitrogen, Carlsbad, CA, USA) respectively according to lipofectamine 2000 instruction (11668–019, Invitrogen, Carlsbad, CA, USA). 48 h after transfection, miR-194 expression and mRNA expression of chemokine receptor type 4 (CXCR4) were measured by RT-qPCR.

In order to study the role of miR-194 in LPS-stimulated macrophages by regulating CXCR4, RAW264.7 cells under LPS exposure were treated with oe-NC or oe-CXCR4 in the presence of miR-194 mimic. Overexpression lentiviruses were purchased from Shanghai Genechem Co., Ltd. (Shanghai, China) and tested according to the instructions. The supernatants of the cells were collected to assess the expression level of inflammatory factors IL-1β, TNF-α and IL-6.

### RT-qPCR

The total RNA was extracted by a RNeasy Mini Kit (Qiagen, Valencia, CA, USA), and cDNA was synthesized using the reverse transcription kit (RR047A, Takara Holdings Inc., Kyoto, Japan) and the miRNA First Strand cDNA Synthesis (Tailing Reaction) kit (B532451–0020, Shanghai Sangon Biotechnology Co., Ltd., Shanghai, China). PCR amplification was performed using the SYBR® Premix ExTaq™ II (Perfect Real Time) kit (DRR081, Takara Holdings Inc., Kyoto, Japan) on a real-time qPCR machine (ABI7500, ABI Company, Foster City, CA, USA). The general reverse primers of miRNA and the forward primers of U6 (internal reference) were provided by the miRNA First Strand cDNA Synthesis (Tailing Reaction) kit, while the other primers were synthesized by Shanghai Sangon Biotechnology Co., Ltd. (Table 1). The Ct value of each well was recorded. Glyceraldehyde-3-phosphate dehydrogenase (GAPDH) was taken as internal reference, and the relative expression of each gene was calculated by the 2^-ΔΔCt^ method.

**Table 1:** The primer sequences of RT-qPCR

Gene	Primer sequences
miR-194	F:5’-ATGGACCTGGGGCCACGAAG-3’
	R:5’- TCTGGCCTGGGAGCGTCG-3’
CXCR4	F:5’-GAGGCCAAGAA ACT GCT G-3’
	R:5’- GCGGTCACAGATGTACCTGTC-3’
U6	F:5'-CAGGGGCCATGCTAAATCTTC-3’
	R:5'-CTTCGGCAGCACATATACTAAAAT-3’
GAPDH	F:5’-CTCATGACCACAGTCCATGCC A-3’
	R:5’-GGATGACCTTGCCCACAGCCT T-3’

Notes: *RT-qPCR* reverse transcription quantitative polymerase chain reaction, *CXCR4* chemokine receptor type 4, *GAPDH* glyceraldehyde-3-phosphate dehydrogenase

### Western blot analysis

The total protein of tissues or cells was extracted using high-efficiency Radio-Immunoprecipitation assay cell lysis buffer (C0481, Sigma-Aldrich Chemical Company, St Louis, MO, USA). A certain amount of total protein was separated by a sodium dodecyl sulfate–polyacrylamide electrophoresis firstly and then transferred onto a polyvinylidene fluoride membrane. After being blocked with 5% BSA for an hour at room temperature, the membrane was immunoblotted with primary antibodies against NF-κBp65 (1:2000, ab16502), phosphor-NF-κBp65 (S536; 1:5000, ab86299), CXCR4 (1:100, ab124824), and GAPDH (1:2500, ab9485) overnight. Afterwards, the membrane was incubated with horseradish peroxidase (HRP)-conjugated secondary goat anti-mouse or goat anti-rabbit immunoglobulin G (IgG, 1:20000, ab205718, Abcam, UK) at room temperature for 1.5 h and visualized by enhanced chemiluminescence detection reagent (NCI4106, Pierce, Rockford, IL, USA). With GAPDH as the internal reference the gray value of each protein band was analyzed by ImageJ 1.48u software (Bio-Rad, Hercules, CA, USA).

### Dual luciferase reporter gene assay

PGLO-CXCR4-wild type (WT) or PGLO-CXCR4-mutant (MUT) were co-transfected with miR-194 mimic or NC mimic into 293 T cells. 24 h after transfection, the cells were lysed and centrifuged for 1 min at 12000 rpm to collect supernatants. The luciferase activity was measured using Dual-Luciferase® Reporter Assay System (Promega Corporation, Madison, WI, USA) according to its manual. The relative luciferase activity was presented as the ratio of firefly luciferase to renilla luciferase.

### Lung wet-to-dry weight (W/D) ratio

After being washed with PBS, the wet weight of the left lung tissues was weighed by an electronic scale and recorded. Then, the tissues were dried at 80 °C for 48 h. Finally, the dry weight of the left lung tissues was weighed and recorded to obtain the W/D ratio.

### Myeloperoxidase (MPO) activity assay

The degree of neutrophil infiltration and MPO activity was determined. After bronchoalveolar lavage, the right lung was removed from the thoracic cavity, dried and stored at − 80 °C. The largest lobe of the right lung was collected, and the supernatant was collected at 4°Cafter being through three cycles of freezing-thawing. The concentration of proteins in the supernatant was determined. A microplate reader (FlexStation 3, Molecular Devices, San Jose, CA, USA) was used to measure the optical density (OD) value at 655 nm after the addition of substrates and catalysts to the supernatant. MPO activity was defined as the change of OD value for per gram of protein per minute.

### Histopathological observation of lung tissues

The lungs were fixed in formalin with 10% neutral buffer for 24 h firstly. Then, the paraffin-embedded lungs were cut into 5 μm sections and stained with hematoxylin and eosin (HE).

### Statistical analysis

SPSS version 19.0 (IBM Corp. Armonk, NY, USA) was used for statistical analysis. The measurement data was presented as mean ± standard deviation, and normality and homogeneity of variance test was conducted. Unpaired *t*-test was used for comparison between two groups of data with normal distribution. The difference was statistically significant at *p* < 0.05.

## Results

### NF-κB plays a damaging role in LPS-induced ALI

To study the role of NF-κB in LPS-induced inflammation in ALI, we used PDTC, which inhibited the activity of NF-κB to see the effects of loss-function of NF-κB in ALI mice. The three groups of mice were treated with LPS only, or combined with either DMSO or PDTC, respectively.

**Fig. 1:**
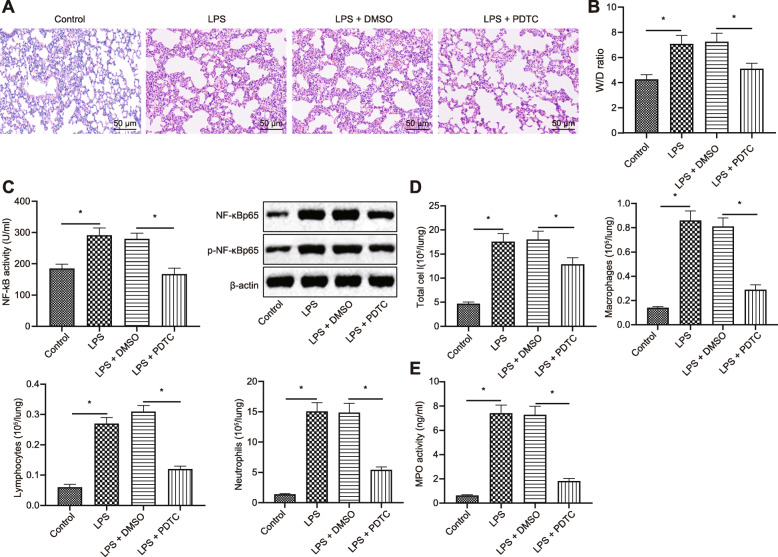
NF-κB aggravates LPS-induced ALI. Mice were treated with PDTC or DMSO in the presence of LPS. **a** Pathological changes of lung tissues in mice by HE staining (200×); **b** W/D ratio of lung tissue in mice; **c** Activity of NF-κB in mice measured by ELISA and western blot analysis; **d** Total cells, macrophages, lymphocytes and neutrophils in BALF of mice; **e** Activity of MPO in different groups of mice. Measurement data were presented as mean ± standard deviation, and data between different groups were compared by unpaired *t*-test (*n* = 12 per group). *, *p* < 0.05

After 24 h of LPS induction, lung tissues were collected for HE staining (Fig. 1a). The results suggested that the structure of alveolar cells in control mice was normal, while the alveolar structure in lungs from LPS and LPS + DMSO-treated mice were disordered, with obvious edema, and a lot of inflammatory cell infiltration. Meanwhile, thickening alveolar septum and alveolar dilatation were observed. Compared with the mice treated with LPS+ DMSO, the lung injury of the mice treated with LPS + PDTC was lighter with partially relieved edema and inflammatory cell infiltration. In addition, the results of W/D of lung tissues showed that compared with the control mice, the ratio of W/D in the mice treated with LPS was higher, while the W/D of mice treated with LPS+ PDTC was lower than that of the mice treated with LPS + DMSO (Fig. 1b). All in all, the inhibition of NF-κB signaling pathway could alleviate LPS-induced acute pulmonary edema.

The activity of NF-κB was detected by ELISA, together with NF-κBp65 expression and the extent of NF-κBp65 phosphorylation measured by western blot (Fig. 1c). It was found that LPS treatment significantly increased the activity of NF-κB, NF-κBp65 expression and extent of NF-κBp65 phosphorylation. Compared with the mice treated with LPS + DMSO, the activity of NF-κB, NF-κBp65 expression, and extent of NF-κBp65 phosphorylation in the mice treated with LPS + PDTC were notably lower, indicating that the activity of NF-κB was remarkably inhibited after treatment with PDTC.

The results of inflammatory cell counting in BALF (Fig. 1d) showed that after LPS induction, the number of inflammatory cells in BALF was increased in varying degrees, which was more pronounced in the mice treated with LPS alone or with DMSO. The number of inflammatory cells in mice treated with LPS + PDTC was significantly lower than that of the mice treated with LPS + DMSO. In addition, the activity of MPO was notably increased in the mice with ALI (Fig. 1e), and mice treated with LPS alone or in the presence of DMSO exhibited more significant trends. The activity of MPO in mice treated with LPS and PDTC was much lower than that of the mice treated with LPS and DMSO, suggesting that the inhibition of NF-κB alleviated the LPS-induced inflammatory responses.

### NF-κB down-regulates miR-194 expression in LPS-induced ALI

**Fig. 2:**
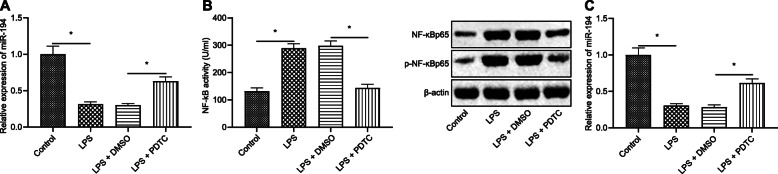
NF-κB down-regulated miR-194 expression in ALI induced by LPS. **a** Expression of miR-194 in mice measured by RT-qPCR; **b** Activity of NF-κB in LPS-treated macrophages detected by ELISA and the expression of NF-κBp65, extent of NF-κBp65 phosphorylation in LPS-treated macrophages assessed by western blot analysis; **c** Expression of miR-194 in LPS-treated macrophages measured by RT-qPCR. The data in the figures were measurements, which were expressed as mean ± standard deviation; data between different groups were compared by unpaired *t*-test. *, *p* < 0.05

miR-194 expression in lung tissues of the different groups of mice were measured by RT-qPCR (Fig. 2a). The results suggested that compared with control mice, miR-194 expression was significantly decreased in mice after the treatment of LPS, but the addition of PDTC, the inhibitor of NF-κB counteracted the decrease of miR-194 expression caused by LPS treatment as the expression level of miR-194 was increased in the mice after being treated with LPS and PDTC. Therefore, these indicated that it was NF-κB that inhibited miR-194 expression in LPS-induced ALI mice.

Next, whether it was NF-κB that down-regulated miR-194 expression was validated with in vitro experiments. After 24 h’ treatment with LPS, the activity of NF-κB in the alveolar macrophages was assessed by ELISA. Besides, NF-κBp65 expression and extent of NF-κBp65 phosphorylation in these LPS treated alveolar macrophages were measured by western blot analysis (Fig. 2b), and miR-194 expression was detected by RT-qPCR (Fig. 2c). The results showed that the activity of NF-κB and miR-194 expression in macrophages was notably decreased after the treatment of LPS. Compared with the macrophages treated with LPS and DMSO, the activity of NF-κB in the mice treated with LPS and PDTC was decreased significantly, while miR-194 expression was increased. Moreover, we used si-NF-κB to treat the macrophages, and the results revealed that si-NF-κB significantly decreased the expression of NF-κB and increased the expression of miR-194, which indicating that NF-κB downregulation may promote miR-194 expression (Supplementary Figure 1). The aforementioned results indicated that NF-κB signaling pathway was activated in LPS-induced ALI, which further down-regulated miR-194 expression.

### CXCR4 is identified to be one of the targets of miR-194

**Fig. 3:**
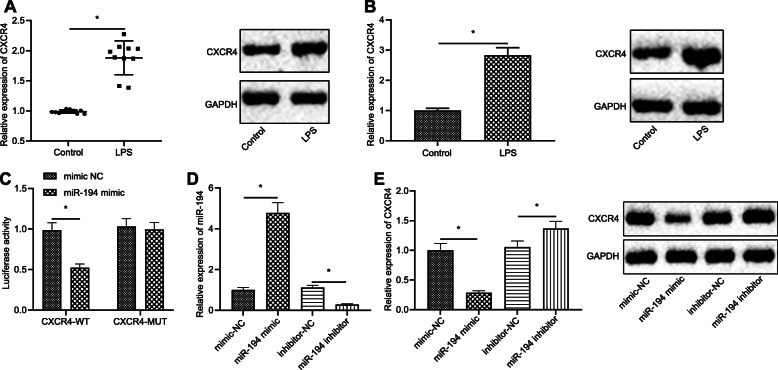
CXCR4 is one of the targets of miR-194. **a** Expression of CXCR4 in LPS-induced mice measured by RT-qPCR and western blot analysis; **b** Expression of CXCR4 in LPS-treated cells detected by RT-qPCR and western blot analysis; **c** Targeting relationship between miR-194 and CXCR4 verified by dual luciferase reporter gene assay; **d** miR-194 expression measured by RT-qPCR; **e** mRNA expression of CXCR4 assessed by RT-qPCR and the protein expression of CXCR4 detected by western blot analysis. The data in the figures were all measurements data, which were presented as mean ± standard deviation; data between different groups were compared by unpaired *t*-test. * indicates *p* < 0.05

Signal transduction of CXCR4 may play a role in affecting the efficient chemotaxis of inflammatory cells. mRNA expression of CXCR4 was detected by RT-qPCR, and protein expression of CXCR4 was assessed by western blot analysis in lung tissues of mice treated with LPS. It was found that CXCR4 expression was significantly increased after LPS treatment (Fig. 3a). Then, after treatment of LPS in macrophages, the mRNA and protein expression of CXCR4 was measured by an RT-qPCR and western blot analysis, respectively. It turned out that CXCR4 expression was notably increased in macrophages with LPS treatment (Fig. 3a).

According to the online prediction website, there might be a strong targeting relationship between miR-194 and CXCR4. Dual luciferase reporter gene assay was used to verify the targeting relationship between miR-194 and CXCR4 in 293 T cells (Fig. 3c). The results showed that compared with mimic NC, miR-194 mimic significantly decreased the luciferase activity in CXCR4-WT (*p* < 0.05), but had no obvious effect on the luciferase activity in CXCR4-MUT (*p* > 0.05). Meanwhile, the transfection efficiency of miR-194 in macrophages, was detected by RT-qPCR (Fig. 3d). It was observed that the miR-194 expression was increased (fold change: 4.5) in cells transfected with miR-194 mimic whereas miR expression was decreased (80%) in cells transfected with miR-194 inhibitor, suggesting the successful transfection. In addition, the mRNA level and protein expression level of CXCR4 in cells with miR-194 successfully silenced or overexpressed was measured by RT-qPCR and western blot analysis, respectively. The results suggested that CXCR4 expression in the cells transfected with miR-194 inhibitor was much higher than those transfected with inhibitor NC. Vice versa, the expression level of CXCR4 was lower in the cells treated with miR-194 mimic than that of cells treated with mimic NC (Fig. 3e). To summarize, all the resulted reflected that miR-194 can negatively regulate CXCR4 expression.

### miR-194 inhibits LPS-induced lung cell inflammation by targeting CXCR4

**Fig. 4:**
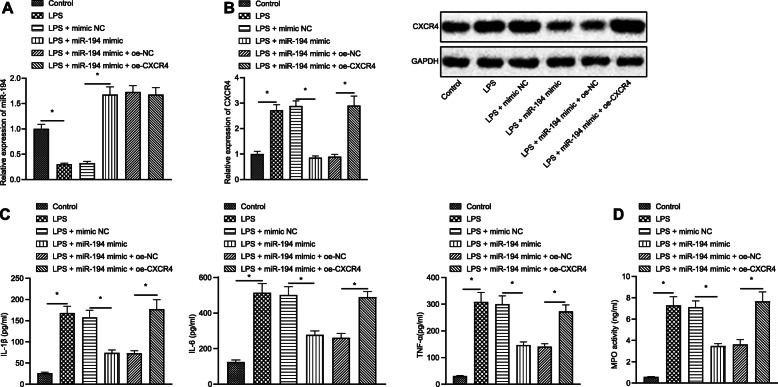
miR-194 retards LPS-induced lung cell inflammation by downregulating CXCR4. Macrophages exposed to LPS were treated with miR-194/NC mimic alone or in the presence of oe-NC/CXCR4. **a** Expression of miR-194 in the transfected cells measured by RT-qPCR; **b** mRNA and protein expression of CXCR4 measured by RT-qPCR and western blot analysis, respectively; **c** Expression of inflammatory factors IL-1β, IL-6 and TNF-α in the transfected cells detected by ELISA; **d** MPO activity in the transfected cells. The data in the figures were all measurements, which were expressed as mean ± standard deviation; data between different groups were compared by unpaired *t*-test. *, *p* < 0.05.

To verify whether miR-194 can affect LPS-induced inflammatory response in cells by targeting CXCR4, macrophages exposed to LPS were treated with miR-194 mimic alone or in the presence of oe-CXCR4. miR-194 expression was detected by RT-qPCR (Fig. 4a). Meanwhile, the mRNA and protein expression of CXCR4 was measured by RT-qPCR and western blot analysis, respectively (Fig. 4b). The results showed that compared with the cells treated with mimic NC, miR-194 expression in cells treated with miR-194 mimic was significantly higher, reflecting the success of transfection, while CXCR4 expression was much lower in cells with miR-194 overexpressed. However, overexpression of CXCR4 together with overexpression of miR-194 (LPS + miR-194 mimic+oe-CXCR4) overcame the down regulating effects of miR-194 on CXCR4 expression.

Then, IL-1β, IL-6 and TNF-α expression in macrophages was measured by ELISA (Fig. 4c). The results revealed that, compared with the cells transfected with mimic NC, IL-1β, IL-6 and TNF-α expression in cells with miR-194 mimic treatment was down-regulated. In addition, IL-1β, IL-6 and TNF-α expression in cells treated with miR-194 mimic and oe-CXCR4 was up-regulated than that in the cells treated with miR-194 mimic and oe-NC. The activity of MPO was measured (Fig. 4d), and it turned out that the activity of MPO in the cells treated with miR-194 mimic was progressively decreased versus the cells treated with mimic NC, while significantly increased after addition of oe-CXCR4 treatment. The results indicated that overexpression of miR-194 could down-regulate CXCR4 expression and significantly reduce the inflammatory response of macrophages, meanwhile overexpression of CXCR4 could abolish the anti-inflammatory effect of miR-194.

### The NF-κB/miR-194/CXCR4 axis regulates LPS-induced inflammatory response of lung cells

**Fig. 5:**
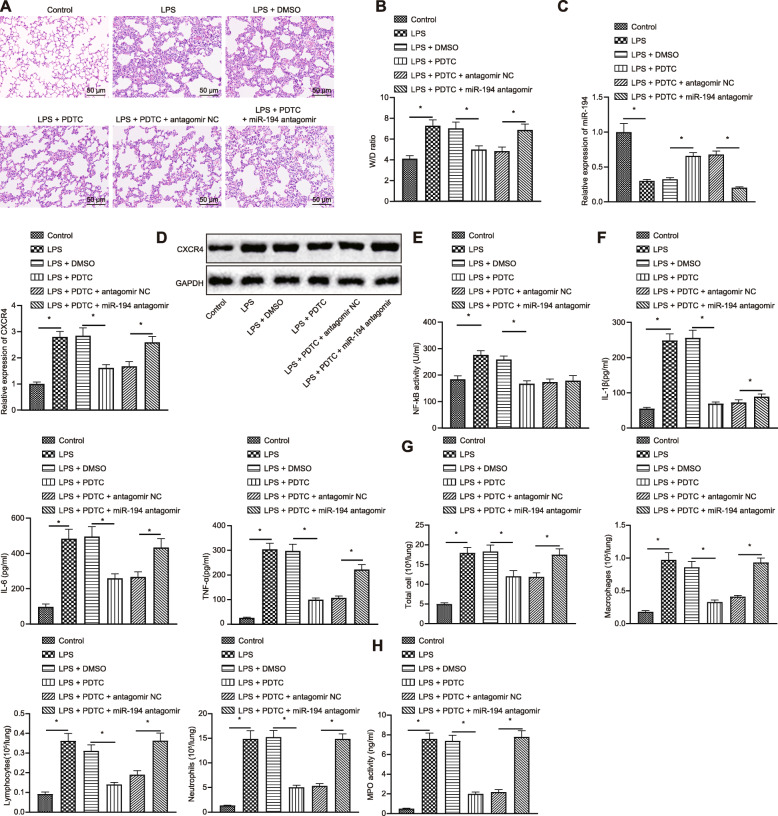
NF-κB aggravates LPS-induced inflammatory response in lung cells by promoting the expression of CXCR4 via down-regulating miR-194. Mice induced with LPS were injected with DMSO/PDTC alone or in the presence of NC /miR-194 antagomir. **a** Pathological changes of lung tissues in mice under different treatments observed by HE staining (200×); **b** W/D ratio of lung tissues in mice under different treatments; **c** miR-149 expression and mRNA expression of CXCR4 measured by RT-qPCR; **d** Expression of CXCR4 at protein level assessed by western blot analysis; **e** Activity of NF-κB detected by ELISA; **f** Expression of IL-1β, IL-6 and TNF-α in BALF of mice measured by ELISA. **g** Number of total cells, macrophages, lymphocytes and neutrophils in BALF of mice; **h** MPO activity of lung tissues in mice under different treatments. The data in the figures were all measurements, which were presented as mean ± standard deviation; data between different groups were compared by unpaired *t*-test. *, *p* < 0.05

In order to clarify the combined role of NF-κB and miR-194 in LPS-induced inflammatory response, we used LPS to induce ALI in mice and to check the inflammatory level while down-regulating the expression of miR-194 or adding the NF-κB inhibitor. Then, the lung tissues were collected for HE staining (Fig. 5a). The results showed that compared with the mice treated with PDTC and antagomir NC, the mice with the treatment of PDTC and miR-194 antagomir had disordered alveolar structure, thickened alveolar wall, obvious edema and thickening in alveolar septum and interstitium. In addition, the results of dry-wet ratio of lung tissues (Fig. 5b) showed that W/D in the mice treated with PDTC and miR-194 antagomir was notably higher than that in the mice treated with PDTC and antagomir NC. Though PDTC, the NF-κB inhibitor exhibited an impactable mitigation effect on the LPS-induced acute pulmonary edema, when the expression of miR-194 was diminished by miR-194 antagomir at the same time, inhibition of miR-194 still reduced this mitigation effect of the NF-κB pathway inhibitor.

Next, the mRNA and protein expression of CXCR4 was measured by RT-qPCR and western blot analysis, respectively (Fig. 5c, d). It was found that CXCR4 expression in the cells from mice treated with PDTC was significantly lower than that in the cells from mice treated with DMSO, while it was remarkably increased in the mice with the treatment of PDTC and miR-194 antagomir compared with those treated with PDTC and antagomir NC.

ELISA revealed that NF-κB activity increased after treated with LPS, but decreased significantly after PDTC treatment (Fig. 5e). Compared with the control mice, the expression of inflammatory factors IL-1β, IL-6 and TNF-α in mice treated with LPS was significantly increased. The expression of inflammatory factors in mice with the treatment of PDTC was much lower than that in the mice treated with DMSO, meanwhile miR-194 antagomir significantly restored the expression of inflammatory factors reduced by PDTC (Fig. 5f). The counting results of inflammatory cells in BALF showed that compared with the mice treated with PDTC and antagomir NC, the number of inflammatory cells and the activity of MPO in the mice treated with PDTC and miR-194 antagomir increased notably (Fig. 5g), and activity of MPO in lung tissues was increased (Fig. 5h). Above all, NF-κB promotes LPS-induced inflammatory response in lung cells by regulating the expression of CXCR4 via down-regulating miR-194.

## Discussion

As a kind of inflammatory lung disease, ALI is manifested as ARDS that seriously affects the morbidity and mortality for critically ill patients [14]. It is predicted that 15 to 20% of patients in ventilation for over 24 h are at risk of ALI, and the mortality rate is as high as 40% [15]. The pathogenesis, and interventions measures of ALI in mice was always clarified by intratracheal administration of LPS [16]. As a component of bacterial cell membrane, LPS can induce the release of many pro-inflammatory cytokines in mice. LPS-induced ALI is characterized by obvious pathological changes including lung edema, alveolar hemorrhage, thickening of alveolar wall and inflammatory cell infiltration [4].

NF-κB, a multipotent regulator of various cellular signaling pathways, participates in certain cell responses to a variety of inflammatory stimuli [17]. Recently, NF-κB has been proved to be involved in the progression of LPS-induced ALI by suppression of oxidative injury in tissue [18]. However, the detail role of NF-κB and its association with miRNAs in ALI remain to be clarified. Therefore, we explored the specific molecular mechanism of NF-κB on LPS -induced ALI.

Acting as important biological regulators, miRNAs are involved in many pathophysiological processes by inhibiting the expression of target genes [19]. Moreover, several studies have demonstrated that miRNAs regulate inflammation and the relevant immune response. For instance, Liu et al. suggested that miR-147 induced by Toll-like receptors stimulation reduced the macrophage inflammatory response by attenuating the expression of TNF-α and IL-6 [20]. Ouimet et al. reported that miR-33 can regulate macrophage inflammation and reduce plaque inflammation by potentiating M2 macrophage polarization and Treg induction [21]. In addition, Li et al. found that apolipoprotein E promotes miR-146a in monocytes and macrophages to inhibit inflammation driven by NF-κB [22]. Furthermore, it was found by Tian et al. that miR-194 attenuated the generation of proinflammatory cytokine in palmitic acid-induced THP-1 Cells [23]. Accordingly, based on our results of RT-qPCR, ELISA and western blot analysis, we founded that in LPS-induced lung cells, the NF-κB was activated, thus inhibiting the expression of miR-194. Similarly, a previous report demonstrated that miR-194 enhanced the LPS-induced the inhibition of cell viability by blocking the NF-κB signaling pathway in WI38 cells [24].

Chemokines are proteins involved in cell migration and inflammatory response, among which CXCR4, a G-protein-coupled receptor, is widely expressed in many tissues, such as endothelial cells, fibroblasts, neutrophils, monocytes, hematopoietic and tissue-committed stem cells [25]. Bai et al. reported that CXCR4 plays a key role in the inflammatory disease process, including lung disease [26]. Besides, the study of Shi and his colleagues suggested that CXCR4 was highly expressed in the lung tissues, and blocking the SDF-1/CXCR4 axis remarkably alleviated the lung injury [27]. Other than these, it was verified that in sepsis-induced ALI, reduced CXCR4+ aged neutrophils by targeting junctional adhesion molecule-C attenuates lung injury and systemic inflammation [28].

Moreover, some studies have suggested that miRNAs can regulate inflammation via modulating CXCR4. For instance, miR-1192 could directly reduce inflammation in vulvovaginal candidiasis by restraining the expression of CXCR4 [29]. Yi et al. reported that overexpression of miR-9-5p in human umbilical vascular endothelial cells can increase proliferation and decrease the apoptosis rate and the expression of inflammatory factors by down-regulating CXCR4 [30]. In order to further clarify the potential mechanism of NF-κB, miR-194 and CXCR4 in LPS-induced ALI, we successfully established 48 ALI mice by spraying LPS into their trachea. Besides, the macrophages RAW264.7 under LPS exposure were treated with miR-194 mimic alone or with oe-CXCR4 to determine the release level of proinflammatory cytokine and the MPO activity. Based on the above experiments, we founded that activated NF-κB signaling pathway in LPS-induced ALI could inhibit the expression of miR-194. However, overexpression of miR-194 could inhibit the CXCR4 expression in macrophages, and down-regulat the expression of inflammatory factors IL-1β, IL-6 and TNF-α, as well as the MPO activity.

## Conclusion

**Fig. 6:**
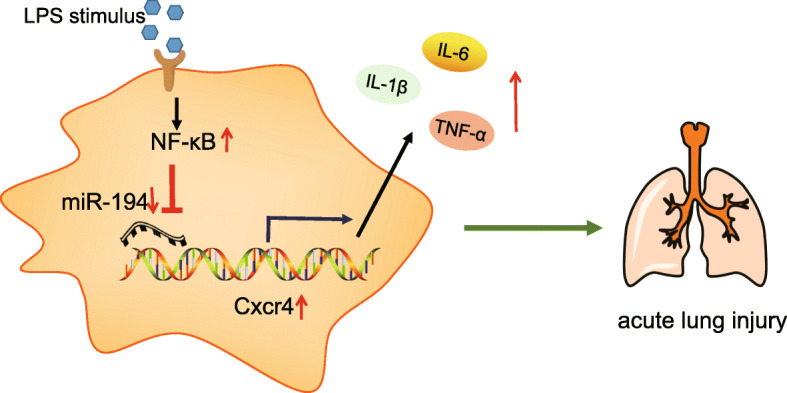
Mechanism of NF-κB on LPS-induced pulmonary inflammatory response via regulating the miR-194-mediated CXCR4. In LPS-induced ALI, NF-κB was activated to down-regulate the expression of miR-194. Consequently, miR-194 negatively regulated CXCR4 was up-regulated to release the inflammatory factors IL-1β, TNF-α and IL-6 which can promote inflammation response

In short, our investigations offered a new insight into the specific mechanism regarding NF-κB in ALI induced by LPS. In this study, we identified that NF-κB can aggravate LPS-induced ALI by regulating the expression of CXCR4 via miR-194 (Fig. 6). Our results suggested that NF-κB functions as a promotive factor in LPS-induced ALI by aggravating pulmonary edema, inflammatory cell infiltration and alveolar dilatation. From what have found in this study, NF-κB may be an innovative and effective therapeutic target for ALI. In further studies, we will expand the sample size of mice models to further verify the NF-κB/miR-194/CXCR4 axis in LPS-induced ALI in mice.

## Supplementary information

**Additional file 1: Figure S1.** NF-κB silencing decreased NF-κB expression and increased miR-194 expression in macrophages. A, mRNA expression of NF-κB by RT-qPCR; B, miR-194 expression by RT-qPCR. The data in the figures were all measurements, which were presented as mean ± standard deviation; data between different groups were compared by unpaired *t*-test. The experiments were repeated 3 times. *, *p* < 0.05.

## Data Availability

The primary data for this study are available from the authors on direct request.

## Abbreviations

ALI: Acute lung injury
NF-κB: Nuclear factor-kappa B
LPS: Lipopolysaccharide
ELISA: Immunosorbent assay
PDTC: Pyrrolidinedithiocarbamate
DMSO: Dimethyl Sulfoxide
CXCR4: Chemokine receptor type 4
RT-qPCR: Reverse transcription quantitative polymerase chain reaction
miRNAs: MicroRNAs
BALF: Bronchoalveolar lavage fluid
PBS: Ehosphate buffer saline
BSA: Bovine serum albumin
ELISA: Enzyme linked immunosorbent assay
HRP: Horseradish peroxidase
WT: wild Type
MUT: Mutant
MPO: Myeloperoxidase
OD: Optical density
